# Sex-specific metabolic profiles of androgens and its main binding protein SHBG in a middle aged population without diabetes

**DOI:** 10.1038/s41598-017-02367-y

**Published:** 2017-05-22

**Authors:** Uwe Piontek, Henri Wallaschofski, Gabi Kastenmüller, Karsten Suhre, Henry Völzke, Kieu Trinh Do, Anna Artati, Matthias Nauck, Jerzy Adamski, Nele Friedrich, Maik Pietzner

**Affiliations:** 1 Institute of Clinical Chemistry and Laboratory Medicine, University Medicine Greifswald, Greifswald, 17475 Germany; 2 Schwerpunktpraxis für Diabetes und Hormonerkrankungen, Erfurt, 99094 Germany; 3 Institute of Bioinformatics and Systems Biology, Helmholtz Zentrum München, Neuherberg, 85764 Germany; 4 Weill Cornell Medical College in Qatar, Education City, Qatar Foundation, Doha, Qatar; 5 Institute for Community Medicine, University Medicine Greifswald, Greifswald, 17475 Germany; 6 DZHK (German Center for Cardiovascular Research), partner site Greifswald, Greifswald, 17475 Germany; 7 DZD (German Center for Diabetes Research), site Greifswald, Greifswald, 17475 Germany; 8 ICB (Institute of Computational Biology), Helmholtz Zentrum München, Neuherberg, 85764 Germany; 9 IEG (Institute of Experimental Genetics), Genome Analysis Center, Helmholtz Zentrum München, Neuherberg, 85764 Germany; 10 Lehrstuhl für Experimentelle Genetik, Technische Universität München, Freising, Weihenstephan 85354 Germany; 11 DZD (German Center for Diabetes Research), site München-Neuherberg, Neuherberg, 85764 Germany; 12 Research Centre for Prevention and Health, Capital Region of Denmark, Glostrup, 2600 Denmark

## Abstract

The role of androgens in metabolism with respect to sex-specific disease associations is poorly understood. Therefore, we aimed to provide molecular signatures in plasma and urine of androgen action in a sex-specific manner using state-of-the-art metabolomics techniques. Our study population consisted of 430 men and 343 women, aged 20–80 years, who were recruited for the cross-sectional population-based Study of Health in Pomerania (SHIP-TREND), Germany. We used linear regression models to identify associations between testosterone, androstenedione and dehydroepiandrosterone-sulfate (DHEAS) as well as sex hormone-binding globulin and plasma or urine metabolites measured by mass spectrometry. The analyses revealed major sex-specific differences in androgen-associated metabolites, particularly for levels of urate, lipids and metabolic surrogates of lifestyle factors, like cotinine or piperine. In women, in particular in the postmenopausal state, androgens showed a greater impact on the metabolome than in men (especially DHEAS and lipids were highly related in women). We observed a novel association of androstenedione on the metabolism of biogenic amines and only a small sex-overlap of associations within steroid metabolism. The present study yields new insights in the interaction between androgens and metabolism, especially about their implication in female metabolism.

## Introduction

Androgens are highly involved in the regulation of metabolism and body composition of men and women. Testosterone (TT), androstenedione (AD), dehydroepiandrosterone and its sulfate (DHEAS) comprise the majority of the systemic circulating androgens^1^. During adulthood, a general decline in serum androgens in both sexes is observed^2, 3^. In men low TT levels were suggested as predictive biomarker for cardiometabolic disease in the elderly^4^ and hence supplementation strategies were discussed but up to now endogenous marker of adequate TT levels are still lacking. We recently showed that low serum levels of TT are associated with increased cardiometabolic morbidity and mortality in men^5^. In contrast to men, associations of androgen levels with increased cardiovascular risk factors like metabolic disorders, including an unfavorable lipid profile or T2DM^6^, are not commonly observed in women^7^ or inconsistent findings are revealed^6^.

Similar to TT, also for the remaining androgens previous studies could not fully clarify the physiological function of these androgens and their role in preservation of health up to now. Nevertheless, previous studies revealed that low DHEAS levels were associated with increased risk of cardiovascular morbidity and mortality in men^1^ and insulin resistance in women^8^. Also with respect to sex hormone-binding globulin (SHGB), the majority of epidemiological studies demonstrated inverse associations with metabolic syndrome (MetS) or T2DM in women^9^ suggesting low SHBG as a potential risk marker for cardiometabolic morbidity, particularly among postmenopausal women^9^. Moreover, in men a meta-analysis revealed a relation of low SHBG level with prevalent and incident MetS^10^, with associations being mainly mediated through hyperglycemia and hypertriglyceridemia. It has to be noted that SHBG not purely defines the content of freely available TT and hence the biological active TT pool but rather raises total steroid hormone content and therefore is a crucial player for steroid hormone action in general^11^.

In general, the role of androgens in metabolism is controversial not only because of the differences in disease associations between the sexes. Whereas a recent whole blood transcriptome analyses^12^ revealed only minor evidence for an interrelation of TT with metabolism, intervention studies^13, 14^ using TT supplementation revealed beneficial metabolic effects at least in males.

Metabolomics could be a suitable tool to bridge the gap between the reported cardiometabolic implications in cross-sectional and intervention studies. Moreover, metabolomic profiles may provide read-outs for the small molecule content in various bio fluids which would help to understand normal androgen metabolism and mediated effects in men and women. Therefore, we aimed to identify small molecular signatures associated with circulating androgens in both sexes using mass spectrometry (MS)-based metabolomics. The selected metabolomics approach covers a wide range of metabolites including amino acids and related derivatives, carbohydrates, energy related metabolites, a comprehensive range of lipid species (including bioactive species like polyunsaturated fatty acids), small peptides, nucleotides but also exogenous derived compounds (i.e. xenobiotics) and a number of up to now unknown compounds. Recent studies already exploit the potential for the identification of new biomarkers of incident type 2 diabetes^15^ or kidney disease^16^ using the same platform. Given the multifactorial implication of diabetes (type 1 and type 2) with androgens, we chose a non-diabetic subsample of 737 subjects from the general population.

## Results

General characteristics for men and women are displayed in Table 1. In general, women exhibited a more beneficial behavior and metabolic status than men including less alcohol consumption, lower WC, less prevalent hypertension, higher high-density lipoprotein (HDL) cholesterol and lower fasting glucose levels or HbA1c. As expected, men and women differed greatly with respect to androgen levels. Most obviously, TT levels were twenty-fold higher in men compared to women (Table 1). Even though to a far lesser extent, DHEAS and AD showed the same trend. Only SHBG showed higher serum levels in women than in men. The correlation pattern between all androgens as well as SHBG is shown in Supplementary Fig. S1.

**Table 1:** General characteristics of the study population.

Characteristics	Men (n = 430)	Women (n = 343)	p*
Age (years)	50 (39; 61)	50 (41; 59)	0.84
Smoking (%)			<0.01
never smokers	31.6	49.0	
former smokers	45.1	26.0	
current smokers	23.3	25.0	
Physically inactive (%)	27.0	27.7	0.82
Alcohol consumption (g/day)	8.6 (3.1; 18.4)	2.6 (0.7; 5.8)	<0.01
Waist circumference (cm)	94 (86; 102)	82 (75; 90)	<0.01
Hypertension (%)	44.0	35.0	0.01
Total cholesterol (mmol/l)	5.3 (4.6; 6.1)	5.5 (4.9; 6.3)	<0.01
HDL cholesterol (mmol/l)	1.27 (1.10; 1.48)	1.54 (1.32; 1.79)	<0.01
Triglycerides (mmol/l)	1.31 (0.92; 1.91)	1.09 (0.78; 1.59)	<0.01
Systolic BP (mmHG)	130.5 (121.0; 140.5)	116.5 (108.0; 128.0)	<0.01
Diastolic BP (mmHG)	78.8 (72.5; 85.0)	75.0 (68.5; 80.0)	<0.01
HbA1c (%)	5.2 (4.9; 5.5)	5.2 (4.8; 5.5)	0.03
Glucose (mmol/l)	5.4 (5.1; 5.8)	5.2 (4.9; 5.6)	<0.01
Number of MetS Components (%):			<0.01
1	19.3	30.6	
2	28.4	27.1	
3	21.6	22.5	
4	6.7	3.2	
ALT (µkatal/L)	0.47 (0.35; 0.65)	0.31 (0.25; 0.43)	<0.01
GGT (µkatal/L)	0.60 (0.45; 0.86)	0.43 (0.36; 0.54)	<0.01
eGFR (ml/min/1.72 m²)	90.9 (81.6; 104.2)	88.4 (75.6; 102.5)	0.03
Testosterone (nmol/l)	17.30 (14.26; 20.53)	0.83 (0.64; 1.06)	<0.01
Androstenedione (nmol/l)	2.80 (2.18; 3.72)	2.48 (1.85; 3.60)	<0.01
DHEAS (mg/l)	1.71 (1.01; 2.52)	1.09 (0.70; 1.54)	<0.01
SHBG (nmol/l)	35.8 (28.5; 45.8)	53.8 (41.4; 73.9)	<0.01

HDL = high density lipoprotein; BP = blood pressure; HbA1c = glycated hemoglobin; ALT = alanine aminotransferase; GGT = γ-glutamyl transpeptidase; eGFR = estimated glomerular filtration rate; DHEAS = dehydroepiandrosterone–sulfate; SHBG = sex hormone-binding globulin; Continuous data are expressed as median (25th percentile; 75th percentile); nominal data are given as percentages. *χ2-test (nominal data) or Mann-Whitney test (interval data) were performed; To convert the values of testosterone from nanomoles per liter to nanograms per deciliter, multiply by 28.82.

### AD

**Figure 1:**
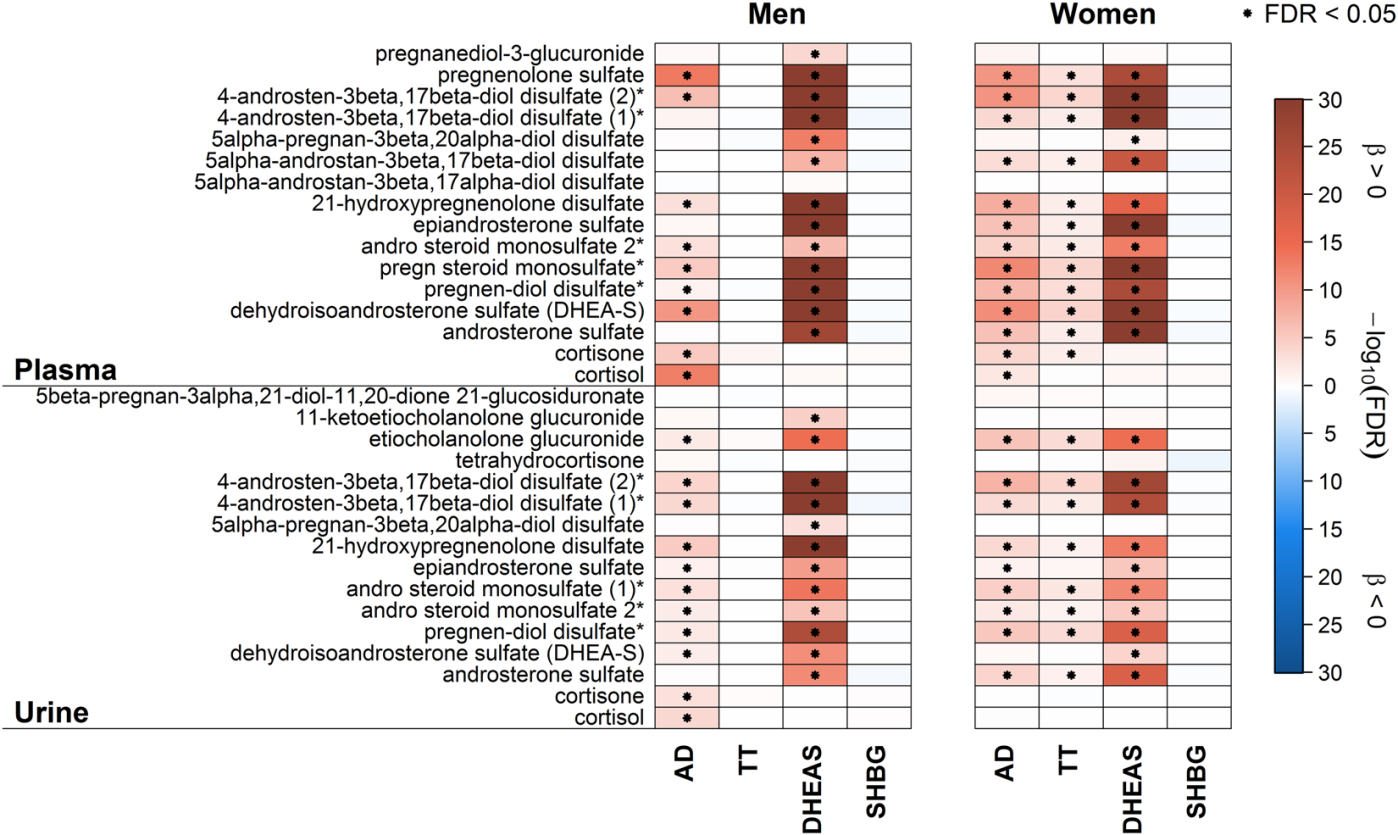
Heatmap of results from linear regression analyses with either androstenedione (AD), testosterone (TT), dehydroepiandrosterone sulfate (DHEAS) or sex hormone-binding globulin (SHBG) as exposure and steroid derivatives in plasma (*upper part*) or urine (*lower part*) as outcome in men (*left panel*) and women (*right panel*), respectively. Orange shading denotes positive and blue shading inverse associations. Dots indicate significant associations by controlling the false discovery rate (FDR) at 5%. Corresponding estimates and FDR values from linear regression analyses can be found in Tables S1–S4. *Metabolites were annotated based on fragmentation spectra.

**Figure 2:**
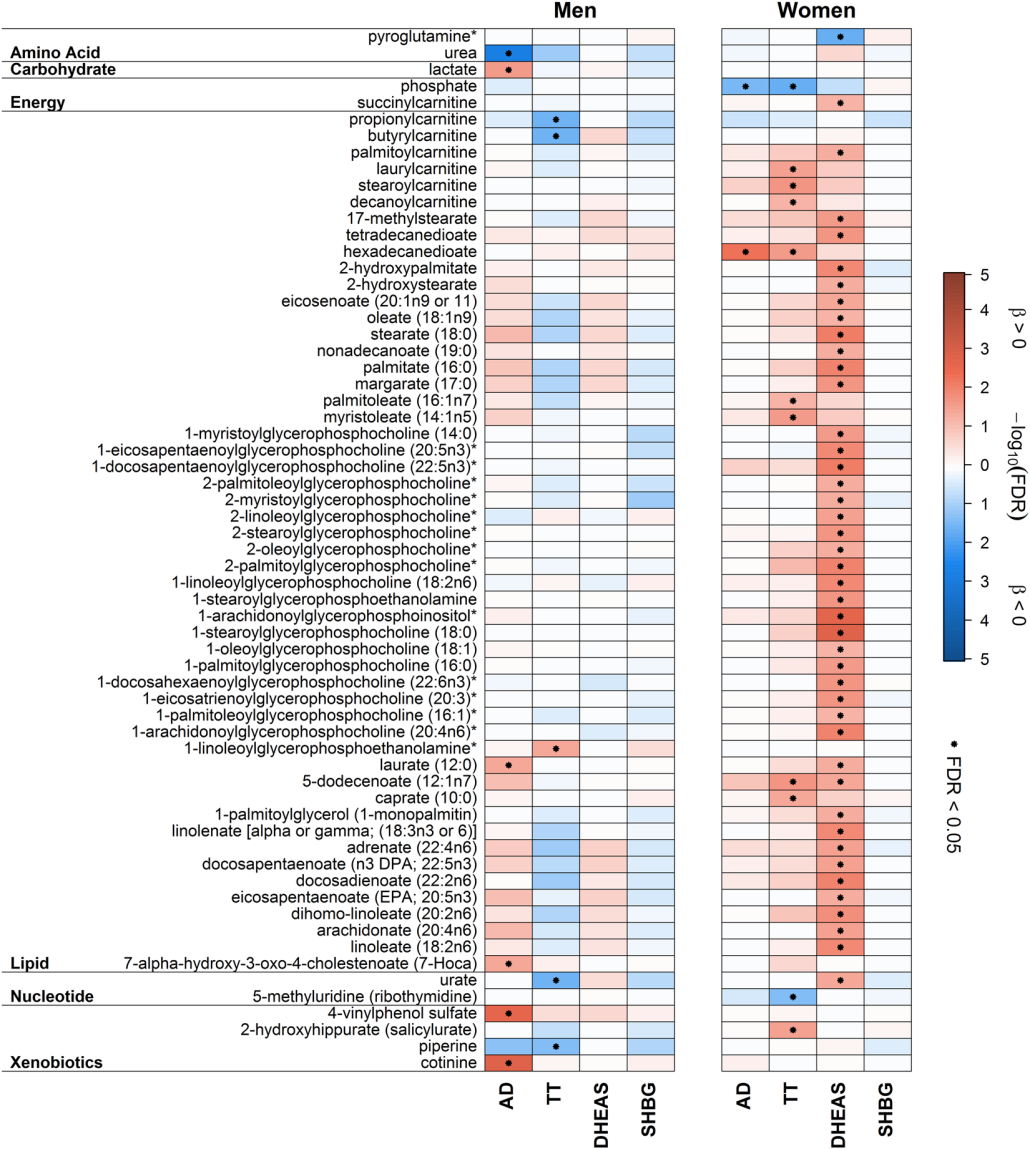
Heatmap of plasma metabolites, excluding steroids and unknown compounds, significantly associated in linear regression analyses with either androstenedione (AD), testosterone (TT), dehydroepiandrosterone sulfate (DHEAS) or sex hormone-binding globulin (SHBG) in men (*left panel*) and women (*right panel*), respectively. Orange shading denotes positive and blue shading inverse associations. Dots indicate significant associations by controlling the false discovery rate (FDR) at 5%. Metabolites were grouped according to physiological entities as denoted on the left. Corresponding estimates and FDR values from linear regression analyses can be found in Tables S1 and S2. *Metabolites were annotated based on fragmentation spectra.

**Figure 3:**
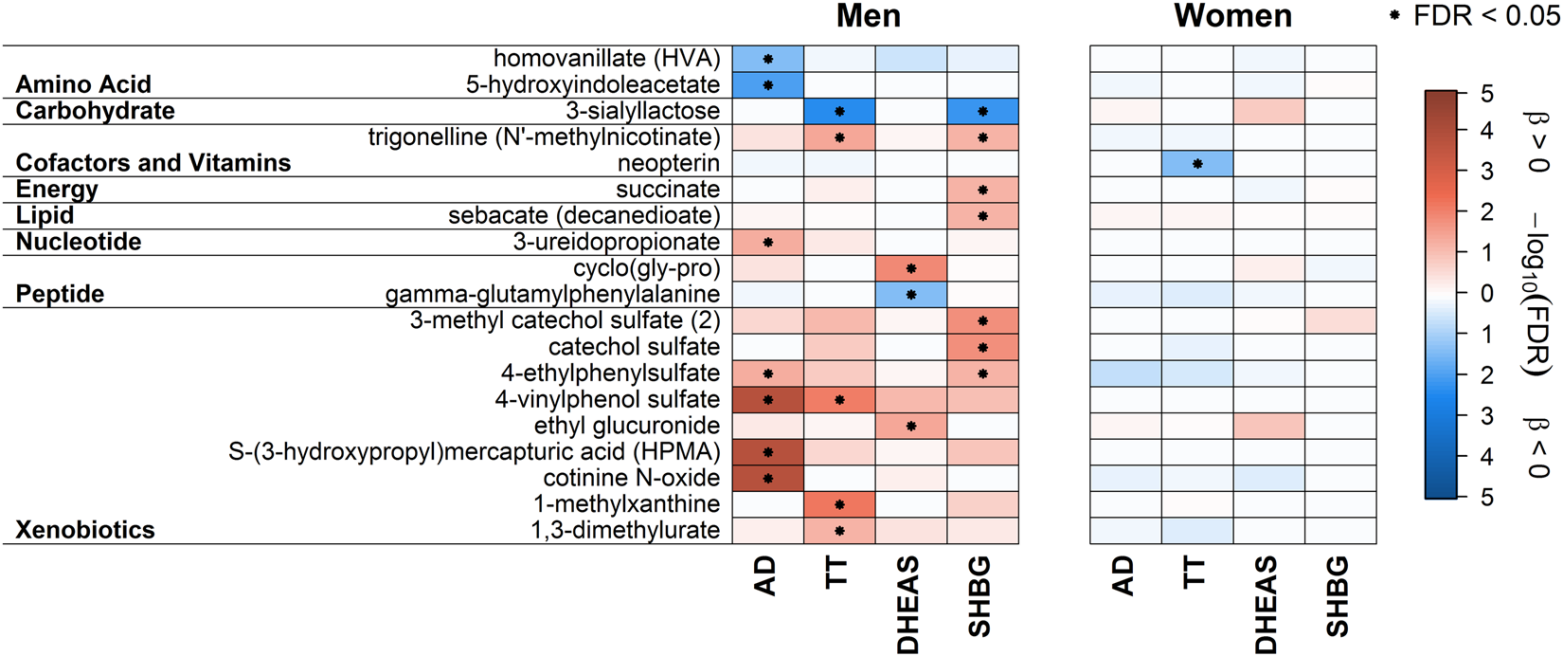
Heatmap of urine metabolites, excluding steroids and unknown compounds, significantly associated in linear regression analyses with either androstenedione (AD), testosterone (TT), dehydroepiandrosterone sulfate (DHEAS) or sex hormone-binding globulin (SHBG) in men (*left panel*) and women (*right panel*), respectively. Orange shading denotes positive and blue shading inverse associations. Dots indicate significant associations by controlling the false discovery rate (FDR) at 5%. Metabolites were grouped according to physiological entities as denoted on the left. Corresponding estimates and FDR values from linear regression analyses can be found in Tables S3 and S4. *Metabolites were annotated based on fragmentation spectra.

In men AD showed significant positive associations with five plasma and five urine metabolites, including two lipids and xenobiotics like cotinine or 4-venylphenol sulfate (4-VP) (Fig. 1) along with two unknown metabolites in plasma and five in urine (Supplementary Table S1 and Fig. S2). In contrast, the amino acid related metabolites urea, 5-hydroxyindoleacetate and homovanillate (Figs 2 and 3) along with seven unknown urine metabolites showed an inverse association (Supplementary Table S3 and Fig. S2). Besides a considerable number of steroid related plasma and urine metabolites (Fig. 1), only plasma hexadecanedioate (Fig. 2), along with two unknown plasma and four urine metabolites (Supplementary Tables S3, S4 and Fig. S2) showed a positive association in women. Inverse associations were found for phosphate (Fig. 2) and two unknown urine metabolites in women (Supplementary Table S4). An overlap in positive associations between men and women was found for nine steroid derivatives in plasma and nine in urine (Fig. 1), respectively. Additionally two unknown plasma and three urine metabolites were shared by the sexes (Supplementary Tables S1–S4 and Fig. S2).

### TT

Among men, none of the investigated steroid related metabolites was associated with TT (Fig. 1). Regarding plasma, most of the associations were invers, including levels of urate, butyrylcarnitine or piperine (Fig. 2). Urine levels of various xenobiotics, like 4-VP, were positively associated (Fig. 3). It has to be noted that additional adjustment for the blood sampling time, to account for a diurnal decline of TT, revealed no obvious difference in the results (Supplemental Fig. S3). Conversely in women, the main part of positively associated plasma metabolites related to lipid metabolism (like free fatty acids (FFA)) (Fig. 2) along with a number of positively associated steroid related plasma metabolites, including DHEAS or sulfates of androsterone, epiandrosterone or pregnenolone (Fig. 1). Serum TT showed an inverse association with four unknown metabolites in plasma and urine in women (Supplementary Tables S3, S4 and Fig. S2). However, none of the TT-associated metabolites in men or women were shared by the opposite sex (Fig. 1).

### DHEAS

The associations between DHEAS and plasma metabolites in men were limited to either steroid derivatives or unknown compounds (Fig. 1 and Supplementary Fig. S2). Urine levels of ethyl glucuronide, the dipeptide cyclo(gly-pro) (Fig. 3) and six unknown metabolites (see Supplementary Table S3) were positively associated with DHEAS whereas gamma-glutamylphenylalanine and two unknown metabolites (Supplementary Table S2) showed inverse associations. In contrast, we detected a greater impact on the plasma metabolome in women in comparison with men (Fig. 1). The main part of the positive associations included more than forty lipids, ranging from lysolipids to FFA (Fig. 1). Additionally urate and twelve unknown metabolites were positively associated (Fig. 1 and Supplementary Table S2). In urine, ten steroid-related metabolites and three unknowns showed an association with DHEAS in women (Fig. 1 and Supplementary Table S4 and Fig. S2).

DHEAS takes a special role in the present analysis, since it is part of the investigated androgens and the set of metabolites. Consequently, a wealth of related steroids showed overlapping associations between men and women with respect to DHEAS (Fig. 1). In detail, twelve steroid derivatives (including sulfates of pregnenolone, androsten, androsterone or epiandrosterone; Fig. 1) and five unknown metabolites in plasma, as well as ten steroid-related metabolites and three unknowns in urine were positively associated with DHEAS in men and women (Supplementary Tables S1–S4 and Fig S2).

### SHBG

Among men, SHBG showed no signficant associations with steroid related metabolites either in plasma or in urine (Fig. 1). For three sulfated compounds (e.g. catechol sulfate, Fig. 3) along with trigonelline (Fig. 3) and seven unknown metabolites (Supplementary Table S3), significant positive associations became obvious in urine. Inverse associations in urine were noted for six unknown metabolites (Supplementary Table S3). Regarding plasma, only two unknown metabolites showed an inverse association with SHBG in men (Supplementary Table S1). No significant associations were found for SHBG in women (Figs 1, 2 and 3 and Supplementary Fig. S2).

### Influence of the menopausal state in women

Stratification of women according to their putative menopausal state revealed stronger associations in postmenopausal than in premenopausal women with respect to plasma metabolites irrespective of the trait under investigation (Supplementary Fig. S4). Except, steroid hormone metabolites were associated to a similar extend in both groups. In contrast, associations between DHEAS and urine metabolites were more apparent in premenopausal women (Supplementary Fig. S5) revealing additional inverse associations with urinary nucleotide conjugates along with a positive association with urinary adenosine. The observation from plasma with respect to AD was also present in urine, whereas the mostly moderate associations towards TT mostly disappeared likely due to limited statistical power.

## Discussion

The present study utilized the ability of metabolomics to broaden the picture of androgen action on metabolism separately for each sex. To investigate the metabolic associations of androgen in the context of a broad spectrum of interfering covariates, we based our analysis on a healthy subsample of middle-aged women and men. Based on plasma and urine metabolite levels determined on an untargeted metabolomics platform, we revealed a remarkably great difference in associated metabolites between men and women, especially regarding urate, intermediates of lipid metabolism, metabolic surrogates of lifestyle factors and several metabolites with unknown identity. In addition, we newly observed that AD putatively interferes with various derivatives of biogenic amino acids, such as metabolites of the neurotransmitters dopamine or serotonin, distinct from TT or DHEAS.

We aimed to investigate the sex-overlap in steroid associated metabolites, which was limited to AD and DHEAS in the present study. The majority of the overlapping metabolites were of steroidogenic origin including precursors and degradation products of steroid metabolism of the adrenal gland (Fig. 1). Although glucuronidation is the major conjugation pathway of androgens in human^17^, we observed a lot of sulfated metabolites, but only few glucuronides. One possible explanation might be the underrepresentation of glucuronidated metabolites detected by the present untargeted LC-MS/MS approach. However, steroids with a 3β-hydroxyl function like pregnenolone, DHEA, androstenediol or androstanediol, are mostly produced by the adrenal gland and are sulfonated to a high extend^17^. The few glucuronides and the urinary androsterone sulfate, a metabolite of TT, are closely linked to TT metabolism and the gonadal function. In conclusion, this observation could be interpreted as the high impact of the metabolism of androgens of the adrenal glands and the lower influence of the gonadal androgen levels. The missing overlap of associated metabolites for TT or SHBG might be not surprising. With respect to TT, men exhibit higher levels through secretion from the testis whereas in women TT secretion is restricted to small amounts out of the ovaries and adrenal glands causing low circulating levels. Moreover, despite its high binding affinity to steroids serum SHBG is affected by a multitude of circumstances, including liver disorders most likely non-alcoholic fatty liver disease (NAFLD) or non-alcoholic steatohepatitis (NASH)^18^. In consequence, differing association patterns between men and women in relation to SHBG likely reflect the divergent metabolic state of both within the present cohort, like a higher amount of alcohol consumption and more prevalent severe MetS in men. To sum up, men and women shared associations in steroid metabolism relaying on DHEAS as an integral metabolite and the steroid-specific associations of TT in women, likely reflect its adrenal origin in women.

The present findings indicate an influence of lifestyle behaviors including smoking, alcohol and coffee consumption on androgen levels. With respect to smoking several associations of AD with metabolites known as surrogates including cotinine, a metabolite of nicotine, 4-VP, 4-ethylphenylsulfate (4-EP) and S-(3-hydroxypropyl)-mercapturic acid were revealed in men^19–21^. This is consistent with previous research reporting higher AD levels in current smokers^22–24^ and an increased AD production after cotinine incubation in rat Leydig cells^25^. Reasons might be a nicotine- and cotinine- mediated inhibition of aromatase^26^ or inhibition of 21- or 11-beta-hydroxylase^23^. English *et al*.^20^ found an association of SHBG with cotinine. Although we did not detect this association in the present study, we observed other biomarkers of smoking to be positively connected with SHBG (trigonelline and 4-EP)^19^. In contrast, one previous study^23^ found no associations between smoking and SHBG. Whereas smoking showed a stronger effect related to AD, TT was related to a urinary signature of coffee consumption, as indicated by markers such as trigonelline and caffeine metabolites such as 1-methylxanthine and 1,3-dimethylurate in men^27^. The influence of coffee on the sexual hormone levels has been controversially discussed in the literature^28, 29^. Previously, Kotsopoulos *et al*.^30^ suggested that the increase in androgen levels is attributable to the inhibition of CYP19 (aromatase) by coffee. Furthermore, we detected a positive association between SHBG and catechol sulfate, which is also increased after coffee intake^31^, and to our knowledge, this association has not previously been described. Taken together, our data suggest that smoking and coffee consumption affects androgen metabolism in men. Interestingly, none of the observed associations became obvious in women. The missing associations might be related to the peri-postmenopausal age of the women in our study. Additionally, a positive association between DHEAS and the urinary ethyl glucuronide level, a well-known biomarker of alcohol consumption^32^, was observed. This finding is in line with a number of studies in men^3, 22^, but not in all^29^. It has to be noted, that none of the other androgens or SHBG showed such an association. This fits to the majority of the literature showing no effect of alcohol intake with respect to TT^28, 29^ or SHBG^24^. The missing association of DHEAS and urinary ethyl glucuronide in women might be likely attributed to the significantly less alcohol consumption within the present cohort.

In men, TT was inversely associated with the commonly used spice piperine, an alkaloid present in black paper extracts^33^. To our knowledge, these associations have not been previously described, but there are reports, which support our findings. An *in*-*vivo* experiment in male mice demonstrated an induced sterility after pepper intake^34^. In part, this decrease in fertility might be attributed to the suppression of the activity of antioxidant enzymes^33^. Moreover, the inhibitory effect of piperine on steroid genesis was even shown with unaltered circulating TT levels^35^. The reported decrease of weight of testes in rats after piperine consumption might be also indicative for a lower androgen stimulus^33, 35^. In summary, the present study suggests transferability of pharmacological findings from rodent models to human males but its degree has to be proven in further studies.

Besides this correlation of androgens with several metabolic surrogates of lifestyle factors, we firstly observed an association between AD and metabolites of dopamine and serotonin, like homovanillate (HVA) and 5-hydroxyindoleacetate (5-HIAA) in men. The neurotransmitter dopamine is degraded by catechol-O-methyl transferase and monoamine oxidase (MAO) enzymes amongst others to HVA, whereas serotonin is broken down only by MAO enzymes^36, 37^. An induced hypogonadism in healthy men did not show any significant changes in HVA or 5-HIAA^38^, whereas increasing production and metabolism of dopamine with increasing TT were observed in an animal model^39^. In addition, it has been reported that sex steroids are involved in the modulation of the neurotransmitter system, nevertheless with conflicting results^40, 41^. Further research is needed to establish the physiological mechanisms behind theses associations as well as to assess a possible causal implication of androgens in neurotransmitter metabolism.

Further, we observed different roles of androgens on plasma urate levels in men and women. In line with Demirbag *et al*.^42^, we observed an inverse association of TT with urate, the final oxidation product of purine metabolism. Urate levels has been associated with erectile dysfunction^43^. Conversely, endogenous TT variation led to no effect on uric acid levels in male volunteers, whereas TT treatment caused a rise in plasma urate level in post-menopausal women^44^. Also a weak codependence between the rise of urate^45^ respectively the fall of TT with increased age^3^ cannot be ruled out safety despite adjustment for age in linear regression analyses. Even though no significant association between TT and uric acid in women became obvious, we observed a positive association to DHEAS as versatile androgenic precursor. In addition, both, DHEAS and urate, are health-related circulating metabolites and thus, this positive association might base on a relatively favorable medical condition. In older subjects higher serum urate levels correlate with better muscle function^46^, whereas lower DHEAS levels are associated with a higher degree of physical disability^47^, along with the already reviewed^48^ increased muscle mass after treatment with DHEAS. Taken together, the sex difference of associated steroid hormones in regards to urate might be based on the differential physiological role of TT and DHEAS in men or women. Whereas TT showed a strong androgenic effect in men, the detailed physiological role of DHEAS in women is rather speculative^49^.

Additionally, a great sex-specific difference of the associated androgens with respect to lipid metabolism became obvious. In contrast to men, among women a profound increase in several lipid species, including lysolipids and FFA, could be observed for higher DHEAS levels and to a minor extend for TT and AD. In human, the effect of androgens on lipid metabolism showed inconsistent results: A study in men reported the expected increase of lipolysis as a result of TT treatment^50^, whereas some investigators did not find any effects of androgens or even an inverse association in both sexes^51, 52^. For women with PCOS, a condition of hyperandrogenism, a link between higher FFA levels and TT has been established^53^. Mai *et al*.^54^ suggested that the FFA-related increase of AD is induced by increased synthesis of its precursor DHEA and not by an amplified production of 17-hydroxyprogesterone. This and the reported elevation of polyunsaturated fatty acids in obese women in consequence of DHEAS treatment^55^ is consistent with our results in women. Despite we and Hernandez-Morante *et al*.^56^ observed an association of DHEAS with lipid metabolism, even the opposite could be reasonable, since lipid infusion in young healthy women decreased DHEAS but not AD clearance^57^. In addition, a competitive effect between steroids and FFA in respect to non-SHBG binding could lead to aligned variations in plasma levels of both^57^.

There is a concentration dependent influence of androgens on the modulation of phospholipase A (PLA), which catalyzes the production of lysolipids in rat testicular cells^58^. In addition, derived lysophospholipids were shown to interfere with 5α-reductase, an enzymes involved in steroid metabolism, resulting mainly in a stimulatory effect^59^, whereas women with PCOS showed decreased levels of the partial hydrolysis products of phospholipids^53^. Besides 5α-reductase, 17β-hydroxysteroid dehydrogenase was affected by, among others, lysophosphatidylcholines resulting in an inhibition^60^, which may account for the detected positive association between DHEAS and lysolipids in women. In summary, the linkage between lysophospholipids and 5α-reductase or androgens merits future work, but the current data implied that PLA and its products lysophospholipids might play important roles as positive or negative effectors in 5α-reductase regulation, dependent on chain length, saturation and tissue.

It has to be noted that stratification of women according to their menopausal state revealed in general stronger associations in postmenopausal women. The most likely explanation might be that the majority of circulating androgens are derived from the adrenal glands. Moreover, this androgen release is no longer influenced by menstrual cycle, whereas in premenopausal women the androgen production will be influenced by ovary production and changes during menstrual cycle. Conformingly, we observed a drop in all androgens after onset of menopause (Supplementary Fig. S7). Therefore in postmenopausal women the metabolic action of androgens in women might be clearer detectable and not be influenced by estrogens or changes during menstrual cycle, leading to stronger associations in our results. Unfortunately, data regarding time point in menstrual cycle are not available so we can’t adjust for this confounding factor in our models.

Another interesting finding of our study was the inverse associations between TT and odd-chain acyl-carnitines in men, which are most likely, derived from branched-chain amino acid (BCAA) catabolism^61^. Patel *et al*.^62^ showed a sex-related heterogeneity in BCAA catabolism, which leads to increased BCAAs and its related metabolites along with a higher insulin resistance in men compared to women^62^. Consistently, an inverse association between TT and insulin resistance was reported in obese men^63^. Our observation may provide a link between a drop in TT, increased BCAA catabolism and impaired insulin sensitivity but this hypothesis has to be proven in appropriate clinical studies.

The evaluation of androgen-associated phenotypes is not only complicated by their differing role between the sexes but also by the fact that the level range matters. Whereas in men immunologic measurements of androgens are quite adequate for measuring TT levels, they must be cautiously evaluated in the lower level range in women for reasons of insufficient sensitivity and accuracy^64^. Consequently, a strength of the present study was the use of LC-MS/MS-based measurements of androgens in both sexes. Furthermore, with regard to metabolomics analyses, evidence for the measurement accuracy of the present metabolomics approach was obtained through excellent correlation of MS-based DHEAS with laboratory results (r = 0.90; see Supplementary Fig. S4). Limitations of the present study include their cross-sectional observational character, which limits insights into metabolic dynamics related to androgen action. Further, the presented associations do not imply causality and replication in independent studies is required.

In conclusion, this study further elucidates the substantial involvement of androgens on the human metabolome and illustrates the benefits of metabolic profiling in biomarker research using untargeted MS-based metabolomics. Our results revealed a great, in part expected, sex-specific mismatch in androgen-associated metabolites in plasma and urine, respectively. Especially associations with urate, lipids, metabolic surrogates of lifestyle factors and several metabolites of unknown identity differed between men and women. Surprisingly androgens showed a higher impact on the metabolome of females, in particular in the postmenopausal state, in comparison to males. With this hypothesis-free approach, we have gained new insights in androgen-related metabolic processes, such as the observed association of AD with the metabolism of biogenic amines. From a clinical point of view, the wealth of associations between DHEAS and lipid metabolism in women might be of special importance in the debate about the increased risk for metabolic and cardiovascular disorders in women associated with altered androgen levels. In contrast, no such supporting information could be derived for TT in men, at least in this almost healthy sample of the general population.

## Methods

### Study Population

The Study of Health in Pomerania (SHIP-TREND) is a second cohort of a population-based research project in West Pomerania, a rural region in northeast Germany^65^. A stratified random sample of 8826 adults aged 20–79 years was drawn from population registries. Sample selection was facilitated by centralization of local population registries in the Federal State of Mecklenburg-West Pomerania. Stratification variables were age, sex and city/county of residence. Baseline examinations were conducted between 2008 and 2012. Out of all invited persons 4420 choose to participate (50.1% response). The study has been approved by the ethics committee of the Ernst-Moritz-Arndt-University of Greifswald and written informed consent was received from all participants prior to the study. The study conformed to the WMA Declaration of Helsinki.

For a subsample of 1000 subjects without self-reported diabetes, plasma and urine metabolomics data were acquired. Subjects with at least one of the following criteria were excluded (overlap existed): completely missing steroid hormone status (N = 3 women) or missing values in covariates (N = 5 men and 5 women), intake of medication influencing serum levels of steroid hormones (ATC: G04CB and L02B, N = 1 men and N = 4 women), history of hysterectomy (N = 89 women) as well as intake of oral contraceptive (ATC: G03A, N = 81 women) or hormone replacement therapy (ATC: G03C, G03D and G03F, N = 31 women). Finally, 430 men and 343 women were available for the present analysis.

### Measurements

Participants’ characteristics and medical histories were recorded using computer-aided personal interviews. Smoking status was categorized as current, former or never smokers. Smoking frequency was recorded as product of daily cigarette consumes and time since smoking onset. Mean daily alcohol consumption was calculated using beverage-specific pure ethanol volume proportions. Subjects who participated in physical training less than two hours a week were classified as physically inactive. Waist circumference (WC) was measured to the nearest 0.1 cm using an inelastic tape midway between the lower rib margin and the iliac crest in the horizontal plane. Height was measured to the nearest 1 cm using a digital ultrasound instrument, and weight was measured using standard digital scales to the nearest 0.1 kg with the subject in light clothing and without shoes. Hypertension was defined by either an increased blood pressure (BP) (systolic BP of ≥140 mm Hg or a diastolic BP of ≥90) or the use of antihypertensive medication (self-report). Dyslipidemia was assumed if one of the following conditions was fulfilled: (1) serum total cholesterol >5.2 mmol/L; (2) low-density lipoprotein (LDL) cholesterol >3.5 mmol/L; (3) HDL cholesterol <1.04 mmol/L or the intake of lipid-modifying medication (ATC code C10AB or C10AD). MetS was defined by three or more of the following five components^66^ using fasting blood samples: (1) abdominal obesity: men WC ≥ 94 cm, women WC ≥ 80 cm; (2) elevated triglycerides: ≥2.3 mmol/l (fasting time < 8 h) or ≥1.7 mmol/l (fasting time ≥8 h) or lipid-modifying medication (ATC code C10AB or C10AD); (3) reduced HDL cholesterol: men < 1.03 mmol/l, women <1.29 mmol/l; (4) elevated blood pressure: ≥130/85 mmHg or self-reported antihypertensive medication or (5) elevated glucose: ≥6.1 mmol/l or diabetic medication (ATC code A10). Menopausal state of the women was categorized using a previously published procedure^67^. Briefly, women not older than 40 years or not older than 60 years but reporting menstrual cycle were classified as premenopausal (N = 161).

Fasting blood samples (≥8 hours) were drawn between 7:00 am and 12:00 pm from the cubital vein of subjects in the supine position and analyzed immediately or stored at −80 °C. Serum levels of TT and AD were measured using liquid chromatography-tandem MS (LC-MS/MS) as reported previously^68^. The standard curve was linear to 50.0 nmol/L and the lower limit of quantitation was 0.25 nmol/L. Intra- and inter-assay coefficients of variation were <10% for both TT and AD over the range 0.3–35 nmol/L. DHEAS and SHBG were measured using a competitive chemiluminescent enzyme immunoassay on an Immulite 2000 analyzer (DPC Biermann GmbH, Bad Nauheim, Germany). Total cholesterol, total triglyceride and serum glucose concentrations were measured by photometry (Dimension VISTA, Siemens Healthcare Diagnostics, Eschborn, Germany). HDL and LDL cholesterol were selectively precipitated and then determined by homogenous assays (Dimension VISTA, Siemens Healthcare Diagnostics, Eschborn, Germany). Serum creatinine levels were measured using the Jaffé method (Dimension VISTA, Siemens Healthcare Diagnostics, Eschborn, Germany). Glycated hemoglobin (HbA1c) was determined by high-performance liquid chromatography (Bio-Rad, Munich, Germany). The eGFR was calculated as: eGFR = 186.3 * serum creatinine^1.154^ * age^0.203^ * (0.702 if female).

### Metabolomics Measurements

Non-targeted metabolomics analysis for metabolic profiling was conducted at the Genome Analysis Center, Helmholtz Zentrum München. A detailed description of metabolite measurements, annotations and data processing is given in the Supplementary Information. Briefly, two separate LC-MS/MS analytical methods were used as previously published^69^ to obtain a broad metabolite spectra in plasma and urine samples in an untargeted manner. Several pre-processing steps were performed which are described in more detail in the supplemental information. Briefly, raw ion counts of metabolites were rescaled with the median of each runday to avoid differences caused by daily variations of platform performances. Metabolites were only kept, if a valid estimation (>3 observations) of the median within a runday was possible. In case of urine, samples were additionally normalized to account for diurnal dilution using probabilistic quotient normalization^70^. Afterwards all metabolites were log2-transformed. Finally, robust multivariate outlier exclusion based on principle component analyses was performed. After pre-processing 475 plasma and 558 urine metabolites remained for the statistical analyses.

### Statistical Analysis

Continuous data are expressed as median (25th; 75th quartile) and nominal data are expressed as percentage. For bivariate analyses, the Mann-Whitney-U test (continuous data) or χ2 test (nominal data) were used to compare women and men. Pearson correlation coefficients were used to display relations between androgens as well as SHBG (Supplementary Fig. S1). Linear regression models were performed to assess the association between androgens as well as SHBG (independent) and plasma as well as urine metabolites (dependent). For this purpose, all independent variables were log-transformed. To avoid spurious results in linear regression analysis, univariate outliers for each metabolite were excluded whenever concentrations exceeded more than three standard deviations from the mean value. All models were performed separately for men and women and adjusted for age, smoking, alcohol consumption and physical activity as well as presence of dyslipidemia or hypertension. Further sensitivity analyses were performed by stratifying women in pre- (N = 161) and postmenopausal (N = 182) women (see Methods and Materials). No such stratification was done in men as the most important androgen, TT, showed no sustainable age effect in our cohort (Supplementary Fig. S6). To account for multiple testing, we adjusted the p-values from regression analyses by controlling the false discovery rate (FDR) at 5% using the Benjamini-Hochberg procedure^71^.

Statistical analyses were performed using SAS version 9.4 (SAS statistical software, version 9.4, SAS Institute, Inc; NC, USA) and R 3.0.1 (R Foundation for statistical computing, version 3.0.1, Vienna, Austria).

## Electronic supplementary material

Supplementary Information
