# Supplementary material for: Sex-specific metabolic profiles of androgens and its main binding protein SHBG in a middle aged population without diabetes
Source: Sci Rep. 2017 May 22;7:2235. doi: 10.1038/s41598-017-02367-y (PMC5440388; doi:10.1038/s41598-017-02367-y)
Supplement: Supplementary file 1 — Supplementary Information [file 41598_2017_2367_MOESM1_ESM.pdf]

SUPPLEMENTARY INFORMATION

Sex-specific metabolic profiles of androgens and its main binding protein SHBG in a middle aged population without diabetes

Uwe Piontek, Henri Wallaschofski, Gabi Kastenmüller, Karsten Suhre, Henry Völzke, Kieu Trinh Do, Anna Artati, Matthias Nauck, Jerzy Adamski, Nele Friedrich, Maik Pietzner

Supplementary Methods

Metabolomics Measurements

Non-targeted metabolomics analysis for metabolic profiling was conducted at the Genome Analysis Center, Helmholtz Zentrum München. Two separate LC-MS/MS analytical methods mere used as previously published, i.e. in positive and in negative ionization modes, were used to detect a broad metabolite panel [1]. In this study, samples were divided into two sets according to the biological matrices of the samples, i.e. plasma and urine. On the day of extraction, samples were thawed on ice. A 100µL of the sample were pipetted into a 2mL 96-well plate. In addition to study samples, a human pooled reference plasma sample (Seralab, West Sussex, United Kingdom) and another pooled reference matrix of each sample set (Seralab, West Sussex, United Kingdom) were extracted and placed in 1 and 6 wells, respectively, of the 96-well plate. These samples served as technical replicates throughout the data set to assess process variability. Beside those samples, 100µL of water was extracted as samples and placed in 6 wells of the 96-well plate to serve as process blanks. Protein was precipitated and the metabolites were extracted with 475µL methanol, containing four recovery standards to monitor the extraction efficiency. After centrifugation, the supernatant was split into 4 aliquots of 100µL each onto two 96-well microplates. The first 2 aliquots were used for LC-MS/MS analysis in positive and negative electrospray ionization mode. Two further aliquots were kept as a reserve. The extracts were dried on a TurboVap 96 (Zymark, Sotax, Lörrach, Germany). Prior to LC-MS/MS in positive ion mode, the samples were reconstituted with 0.1% formic acid (50µl for plasma, 100µl for urine). Whereas samples analyzed in negative ion mode were reconstituted with 6.5mM ammonium bicarbonate (50µl for plasma, 100µl for urine), pH 8.0. Reconstitution solvents for both ionization modes contained internal standards that allowed monitoring of instrument performance and also served as retention reference markers. To minimize human error, liquid handling was performed on a Hamilton Microlab STAR robot (Hamilton Bonaduz AG, Bonaduz, Switzerland). LC-MS/MS analysis was performed on a linear ion trap LTQ XL mass spectrometer (Thermo Fisher Scientific GmbH, Dreieich, Germany) coupled with a Waters Acquity UPLC system (Waters GmbH, Eschborn, Germany). Two separate columns (2.1 x 100 mm Waters BEH C18, 1.7 µm particle-size) were used

either for acidic (solvent A: 0.1% formic acid in water, solvent B: 0.1% formic acid in methanol) and or for basic (A: 6.5mM ammonium bicarbonate, pH 8.0, B: 6.5mM ammonium bicarbonate in 95% methanol) mobile phase conditions, optimized for positive and negative electrospray ionization, respectively. After injection of the sample extracts, the columns were developed in a gradient of 99.5% A to 98% B over an 11 min run time at 350µLl/min flow rate. The eluent flow was directly run through the ESI source of the LTQ XL mass spectrometer. The mass spectrometer analysis alternated between MS and data-dependent MS/MS scans using dynamic exclusion and the scan range was from 80-1000 m/z. Metabolites were identified by Metabolon, Inc. from the LC-MS/MS data by automated multiparametric comparison with a proprietary library, containing retention times, m/z ratios, and related adduct/ fragment spectra[2]. Identification criteria for the detected metabolites are described in Evans *et al.*[1]. Quality control methods and normalization of metabolite levels are explained in detail in the supplement.

Metabolomics Measurements: Quality Control and Normalization of Metabolite Levels

To correct for daily variations of platform performance, the raw ion count of each metabolite was rescaled by the respective median value of the run day. Valid estimation of the median was ensured by keeping only metabolites with at least three measured values on more than the half of the run days. This procedure resulted in 475 and 558 metabolites for plasma and urine, respectively, available for the present analysis. 263 metabolites were measured in both bio fluids. We chose probabilistic quotient normalization (PQN) [3] to account for diurnal variation of urine samples, since this procedure was shown to be superior to the common creatinine scaling. For this purpose we calculated a mean-pseudo-spectrum depending on metabolites with measurements for all participants (131 urine metabolites). Subsequently, we calculated a dilution factor as the median quotient between the reference spectrum and each sample. Of note, urine creatinine and the estimated dilution factor were highly correlated ($r=0.91$, $p<0.001$) within the present study sample. Afterwards all metabolite levels were $\log_2$-transformed. Separately for plasma and urine samples we performed multivariate outlier detection using an algorithm proposed by Filzmoser *et al.* [4] as implemented in the *pcout* function within the R package *mvoutlier*. The algorithm provides an outlier score for each sample based on a weighted combination of location and scatter estimations using principle component analysis and the Mahalanobis distance on a robustly scaled data matrix. The default parameters were used for the identification process, except the critical value for the location outliers was set to 4, as it corresponds to a 4 SD exclusion criteria. The minimum score was used as cut-off for outlier identification. As a result 13 and 8 samples from plasma and urine were excluded, respectively.

References

1 Evans, A. M., DeHaven, C. D., Barrett, T., Mitchell, M. & Milgram, E. Integrated, nontargeted ultrahigh performance liquid chromatography/electrospray ionization tandem mass spectrometry platform for the identification and relative quantification of the small-molecule complement of biological systems. *Analytical chemistry* **81**, 6656-6667, doi:10.1021/ac901536h (2009).

2 Lawton, K. A. *et al.* Analysis of the adult human plasma metabolome. *Pharmacogenomics* **9**, 383-397, doi:10.2217/14622416.9.4.383 (2008).

3 Dieterle, F., Ross, A., Schlotterbeck, G. & Senn, H. Probabilistic quotient normalization as robust method to account for dilution of complex biological mixtures. Application in 1H NMR metabonomics. *Analytical chemistry* **78**, 4281-4290, doi:10.1021/ac051632c (2006).

4 Filzmoser, P., Maronna, R. & Werner, M. Outlier identification in high dimensions. *Computational Statistics and Data Analysis* **52**, 1694-1711 (2008).

Figures

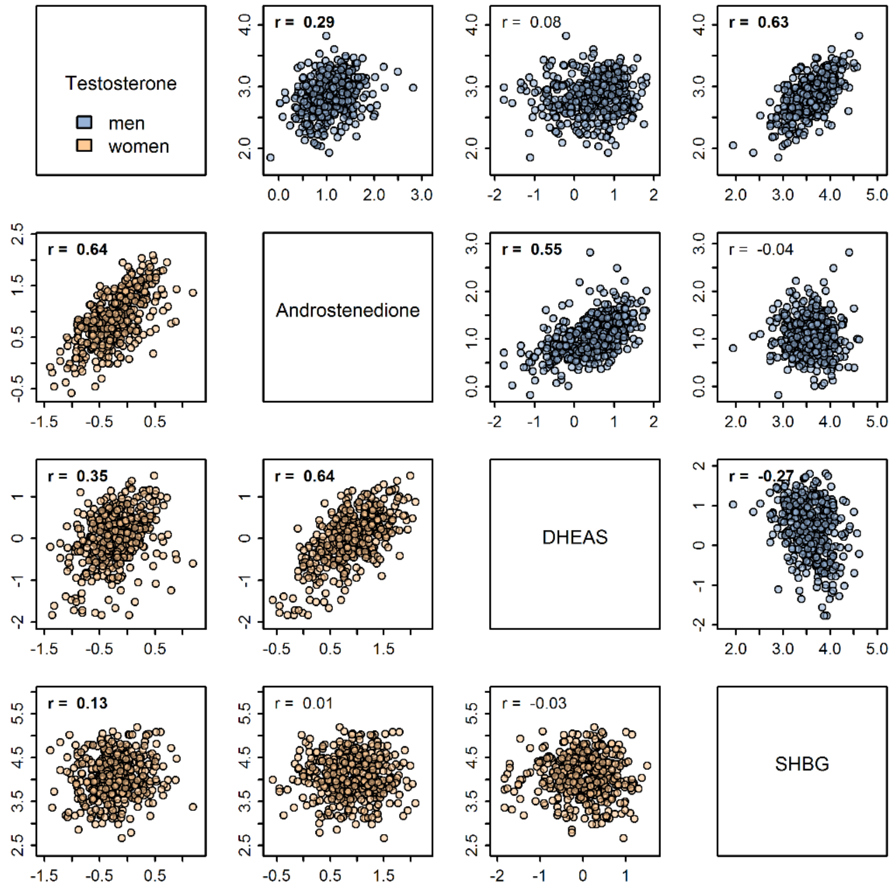

Supplementary Figure S1 Scatterplots showing the pairwise correlations for serum levels of testosterone, androstenedione, dehydroepiandrosterone sulfate (DHEAS) and sex hormone-binding globulin (SHBG) separately for men (*blue: upper triangle*) and women (*orange: lower triangle*). All variables were log-transformed for this purpose and corresponding Pearson correlation coefficients are given (significance is indicated by bold characters).

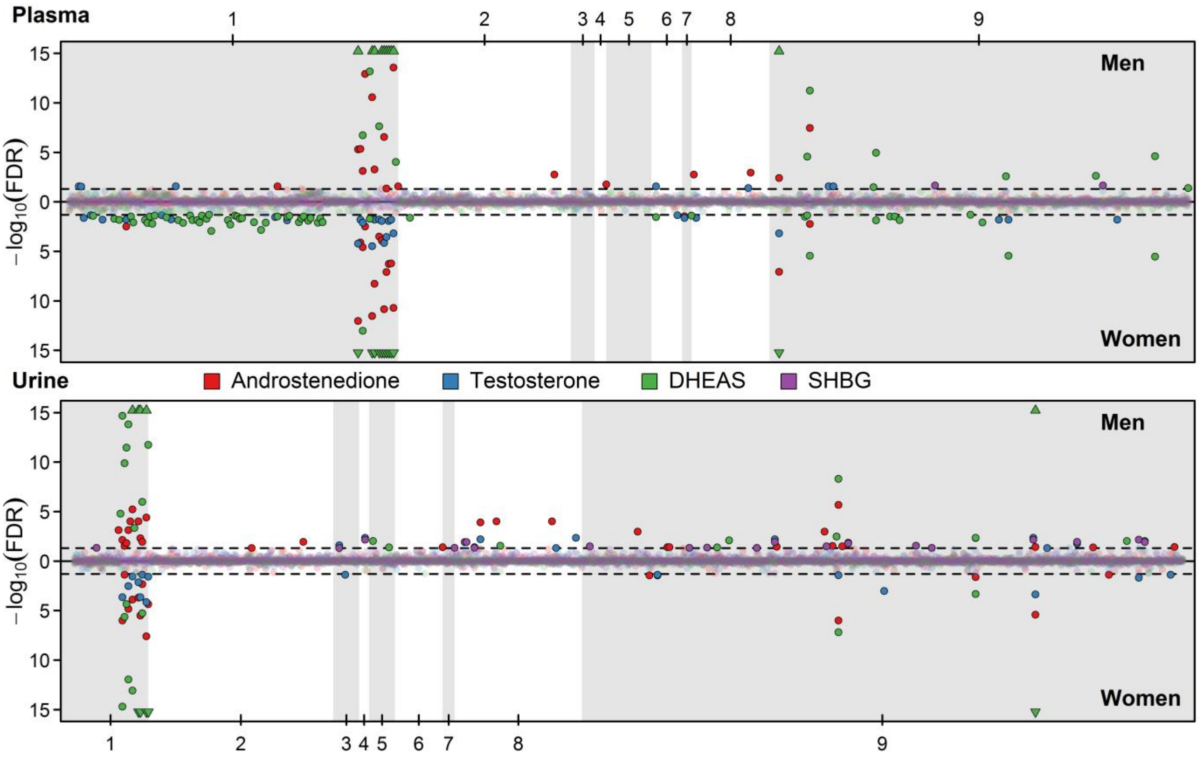

Supplementary Figure S2 Adjusted p-values (false discovery rate: FDR) from univariate linear regression analysis of plasma (*upper panel*) or urine metabolites (*lower panel*) associated with serum levels of testosterone, androstenedione, sex hormone-binding globulin (SHBG) and dehydroepiandrosterone sulfate (DHEAS) in men and women. Within each panel the dotted lines denote a FDR < 0.05, representing the significance threshold. Metabolites were grouped according to the following classes: 1) lipids 2) amino acids 3) cofactors and vitamins 4) carbohydrates 5) peptides 6) nucleotides 7) energy 8) xenobiotics and 9) unknown identity. Metabolites marked with a triangle exceeded the plotting range.

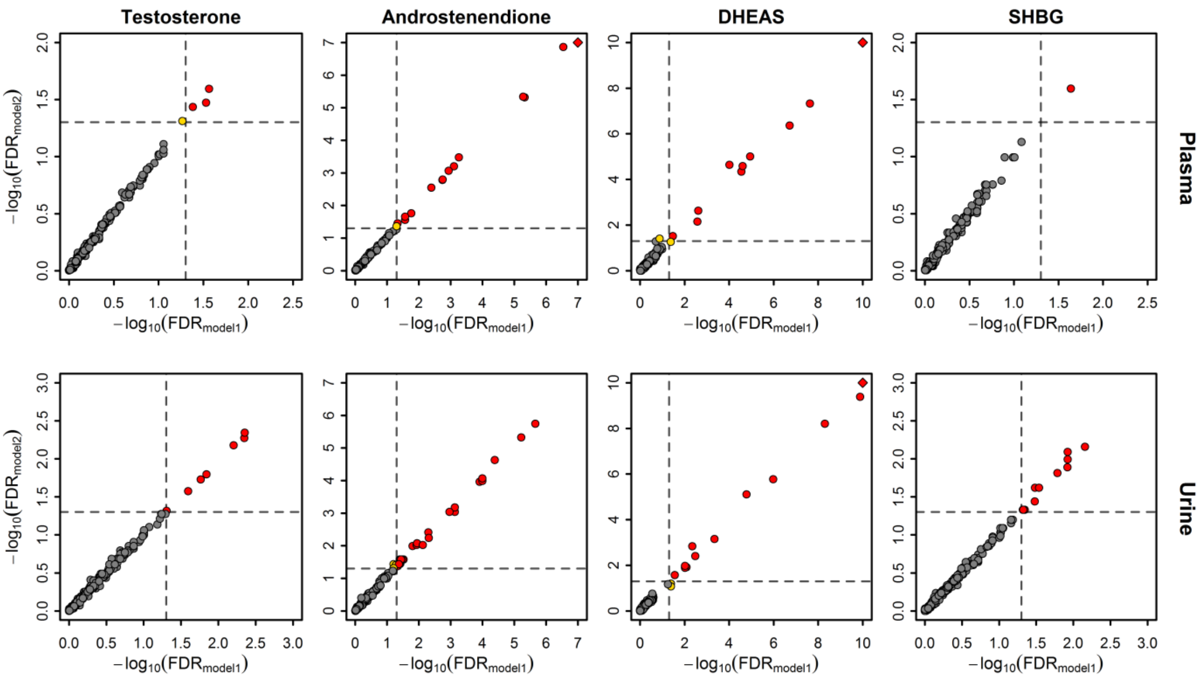

Supplementary Figure S3 Comparison of adjusted p-values (false discovery rate, FDR) from two different linear regression models with either testosterone, androstenedione, dehydroepiandrosterone sulfate (DHEAS) or sex hormone-binding globulin (SHBG) as exposure and plasma (upper panel) and urine metabolites (lower panel) as outcome in men. The first model (model1) was adjusted for age, smoking, alcohol consumption and physical activity as well as presence of dyslipidemia or hypertension. The second model (model2) further contained blood sampling time as covariate. Red dots indicate significant metabolites in both models whereas yellow dots indicate metabolite significantly associated in one of the models.

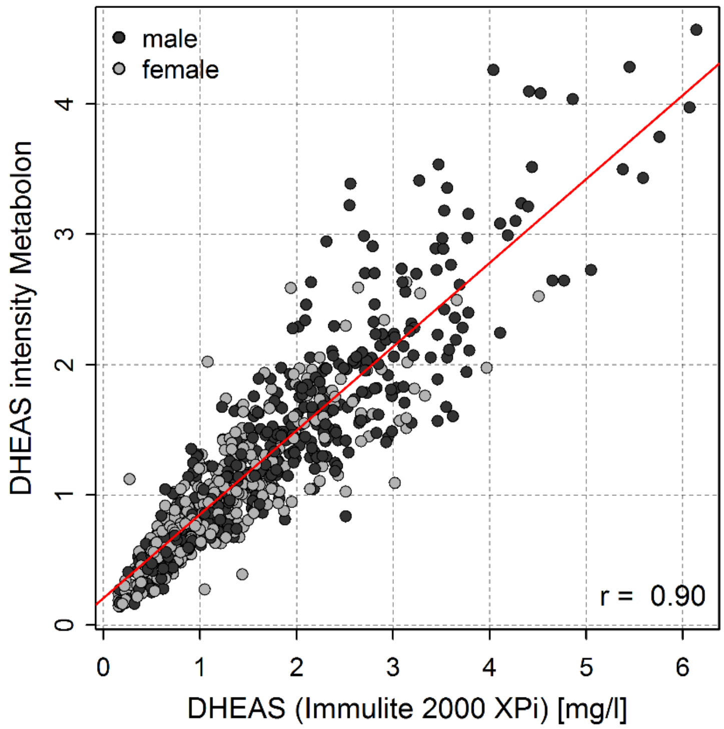

Supplementary Figure S3 Scatterplot comparing measurements of dehydroisoandrosterone sulfate (DHEAS) either with immunoassay (Immulite 2000 analyzer, DPC Biermann GmbH, Bad Nauheim, Germany) or by Metabolon Inc.

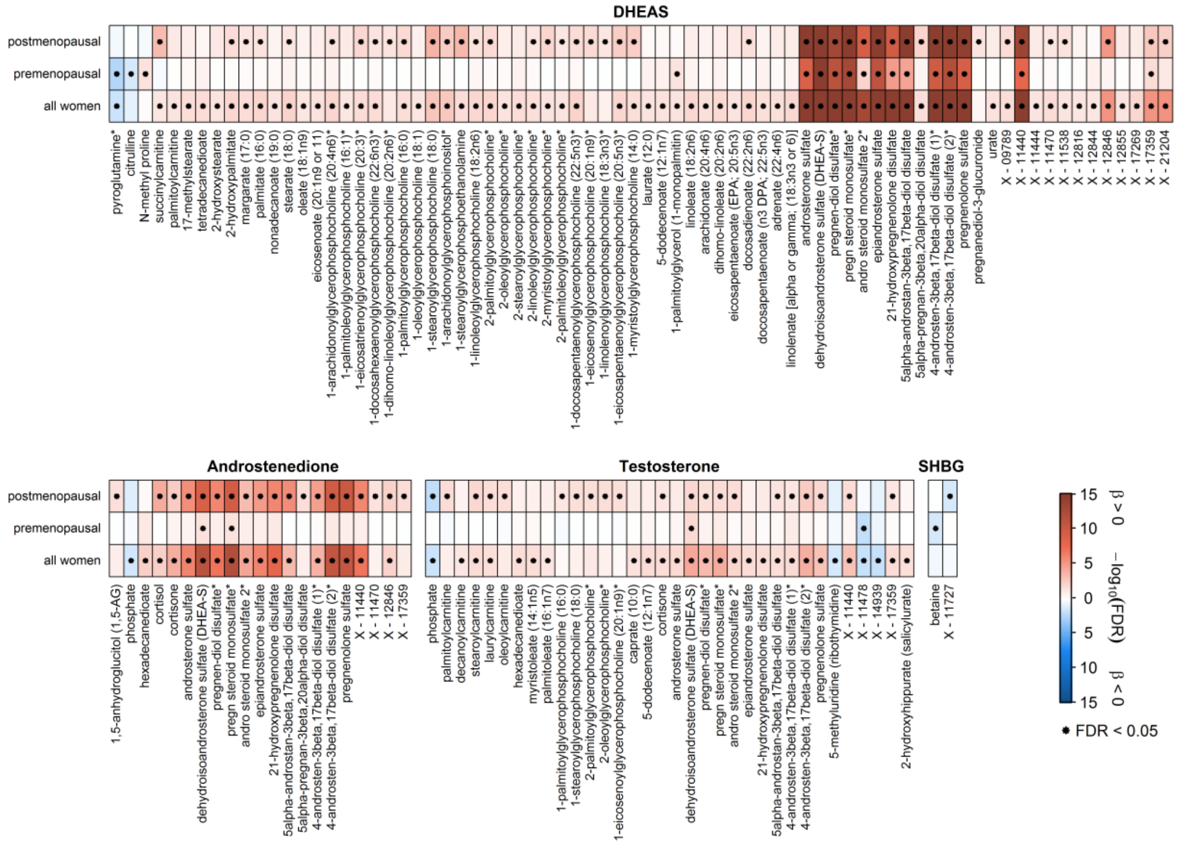

Supplementary Figure S4 Heatmaps of results from linear regression analyses with either dehydroepiandrosterone sulfate (DHEAS; upper panel), androstenedione, testosterone or sex hormone-binding globulin (SHBG; all lower panel) as exposure and **plasma** metabolites as outcome. Each row of a heatmap represents a specific population: 1) all women, 2) premenopausal women and 3) postmenopausal women. Orange shading denotes positive and blue shading inverse associations. Dots indicate significant associations by controlling the false discovery rate (FDR) at 5%. *Metabolites were annotated based on fragmentation spectra

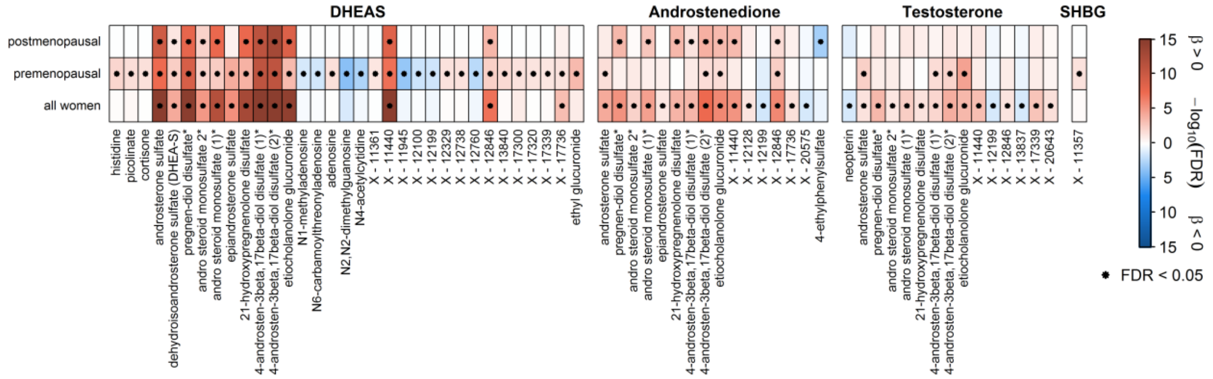

Supplementary Figure S5 Heatmaps of results from linear regression analyses with either dehydroepiandrosterone sulfate (DHEAS), androstenedione, testosterone or sex hormone-binding globulin (SHBG) as exposure and **urine** metabolites as outcome. Each row of a heatmap represents a specific population: 1) all women, 2) premenopausal women and 3) postmenopausal women. Orange shading denotes positive and blue shading inverse associations. Dots indicate significant associations by controlling the false discovery rate (FDR) at 5%. *Metabolites were annotated based on fragmentation spectra

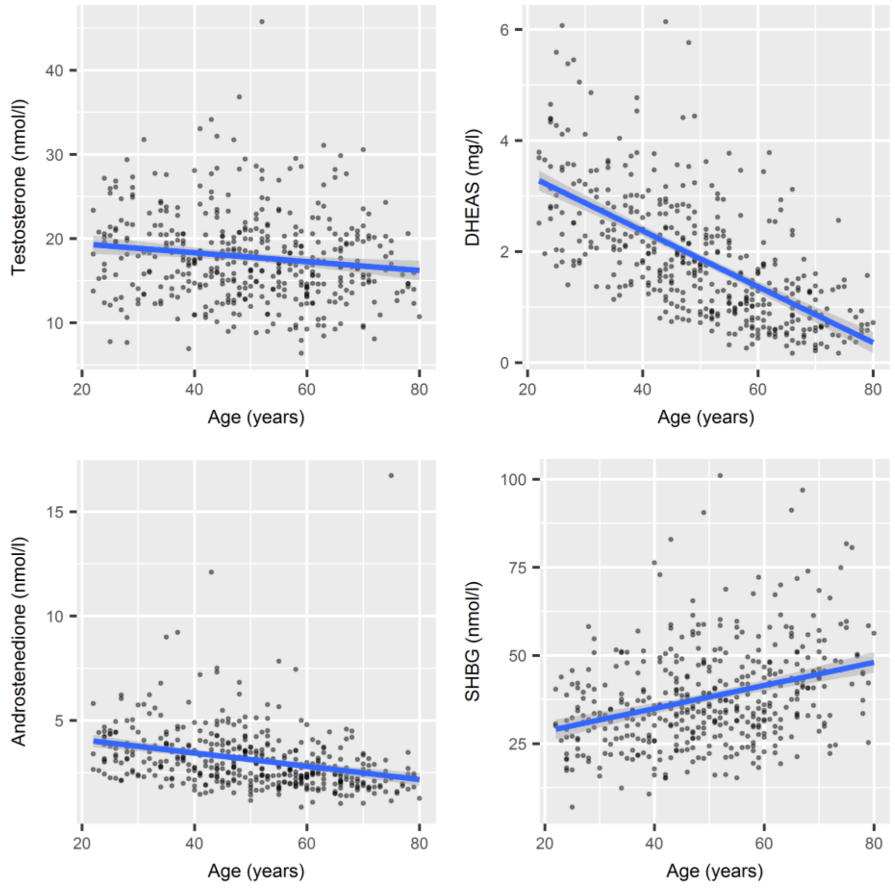

Supplementary Figure S6 Age dependency of testosterone, androstenedione, dehydroepiandrosterone sulfate (DHEAS), and sex hormone-binding globulin (SHBG) in men. Blue lines indicate a linear regression fit with 96%-confidence interval.

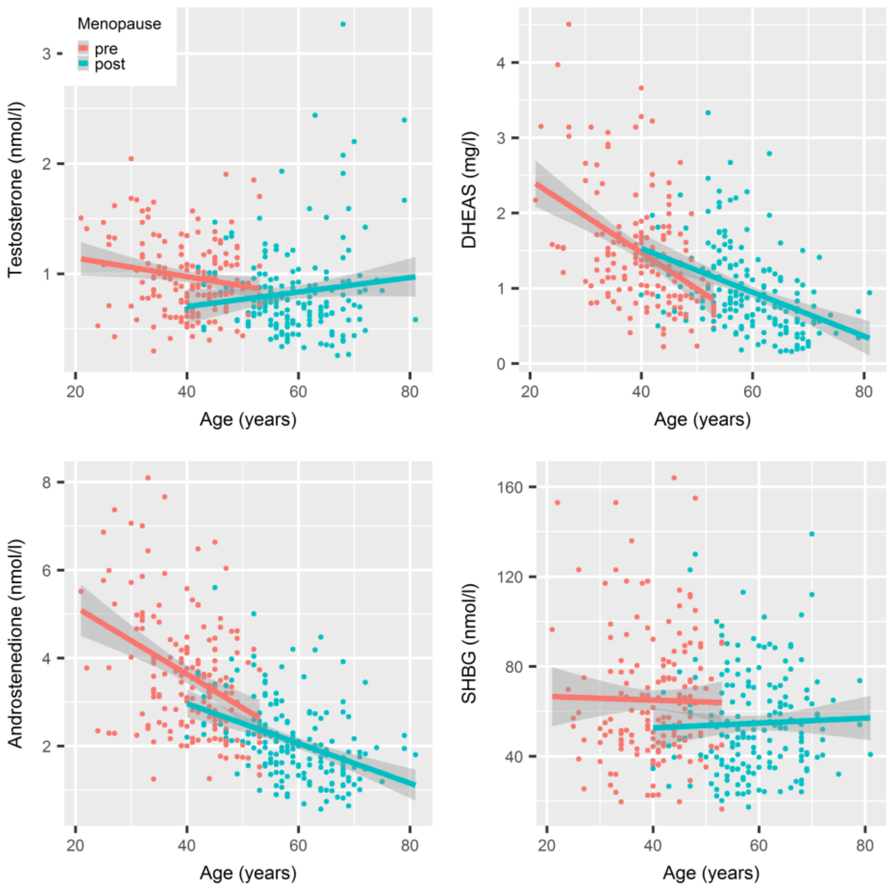

Supplementary Figure S7 Age dependency of testosterone, androstenedione, dehydroepiandrosterone sulfate (DHEAS), and sex hormone-binding globulin (SHBG) in women stratified by the menopausal state (pre = orange; post = blue). Lines indicate a linear regression fit with 96%-confidence interval.
